# Validation of the relative biological effectiveness of active-energy scanning carbon-ion radiotherapy on a commercial treatment planning system with a microdosimetic kinetic model

**DOI:** 10.1186/s13014-023-02267-8

**Published:** 2023-05-17

**Authors:** Weiwei Wang, Wei Sun, Hao Shen, Jingfang Zhao

**Affiliations:** 1 Department of Medical Physics, Shanghai Proton and Heavy Ion Center, Fudan University Cancer Hospital, Shanghai Key Laboratory of Radiation Oncology (20dz2261000), Shanghai Engineering Research Center of Proton and Heavy Ion Radiation Therapy, 4365 Kangxin Road, Pudong District, Shanghai, 201315 China; 2 Institute of Modern Physics, Applied Ion Beam Physics Laboratory, Fudan University, Shanghai, 200433 China; 3 Department of Medical Physics, Shanghai Proton and Heavy Ion Center, Fudan University Cancer Hospital, 270 Dongan Road, Xuhui District, Shanghai, 200032 China

**Keywords:** CIRT, MKM, Validation, RayStation, Active scanning

## Abstract

**Background:**

The study objective was to validate the relative biological effectiveness (RBE) calculated by the modified microdosimetric kinetic model in RayStation (Ray-MKM) for active-energy scanning carbon-ion radiotherapy.

**Methods:**

The Ray-MKM was benchmarked using a spread-out Bragg-peak (SOBP) plan, which was suggested in literature from the National Institute of Radiobiological Science (NIRS) in Japan. The residual RBE differences from the MKM at NIRS (NIRS-MKM) were derived using several SOBP plans with different ranges, SOBP widths, and prescriptions. To investigate the origins of the differences, we compared the saturation-corrected dose-mean specific energy $Z_{1D}^{*}$ of the aforementioned SOBPs. Furthermore, we converted the RBE-weighted doses with the Ray-MKM to those with local effect model I (LEM doses). The purpose was to investigate whether the Ray-MKM could reproduce the RBE-weighted conversion study.

**Results:**

The benchmark determined the value of the clinical dose scaling factor, $F_{clin}$, as 2.40. The target mean RBE deviations between the Ray-MKM and NIRS-MKM were median: 0.6 (minimum: 0.0 to maximum: 1.69) %. The $Z_{1D}^{*}$ difference in-depth led to the RBE difference in-depth and was remarkable at the distal end. The converted LEM doses from the Ray-MKM doses were comparable (the deviation being − 1.8–0.7%) to existing literature.

**Conclusion:**

This study validated the Ray-MKM based on our active-energy scanning carbon-ion beam via phantom studies. The Ray-MKM could generate similar RBEs as the NIRS-MKM after benchmarking. Analysis based on $Z_{1D}^{*}$ indicated that the different beam qualities and fragment spectra caused the RBE differences. Since the absolute dose differences at the distal end were small, we neglected them. Furthermore, each centre may determine its centre-specific $F_{clin}$ based on this approach.

## Background

Carbon-ion radiotherapy (CIRT) has been used to treat human cancer at the National Institute of Radiological Sciences (NIRS) in Japan since 1994 [1, 2]. Years of clinical applications have shown that CIRT is a highly promising treatment modality for several cancer types [3]. However, the accurate modelling of carbon-ion relative biological effectiveness (RBE) is challenging. The physical parameters, including linear energy transfer (LET) and absorbed dose, vary along the beam paths [4]. Therefore, a biophysical model is necessary for CIRT.

To date, three biophysical models have been used in clinical CIRT. They are the Kanai model [5], the modified microdosimetric kinetic model (MKM) [6–8], and the local effect model I (LEM) [9]. The Kanai model was designed for CIRT with a range shifter scanning (RS) beam, i.e., the Heavy Ion Medical Accelerator at Ciba (HIMAC) [1]. The RS beam or the broad beam shifts the Bragg-peak depth by inserting range shifters. This model assumes the human salivary gland (HSG) tumour cell to represent the moderate radiosensitivity of human tumours to carbon ions, and uses empirical HSG tumour cell data, which are the linear quadratic (LQ) parameters of HSG tumour cells as a function of the linear energy transfer (LET) of monoenergetic carbon-ion beams, i.e., LET-α and LET-β tables, to calculate the biological RBE for the mixed beam. The biological RBE is further rescaled to clinical RBE by using the NIRS fast neutron experience [5]. Since 2011, the NIRS has adopted a hybrid scanning (HS) beam [10, 11]. The HS beam uses several beam energies in conjunction with the range shifter plates to shift the Bragg-peak depth. Compared to the RS beam, the HS beam does not require apertures or compensators. Meanwhile, compared to active-energy scanning (ES), i.e., where the Bragg-peak depth is shifted by changing the beam-extraction energy of the synchrotron [12], the HS beam offers faster delivery. The MKM was developed based on the Kanai model but dedicated to the HS beam at the NIRS (we call it the NIRS-MKM hereafter) [8] to correct the RBE overprediction by the Kanai model, e.g., the RBE at the distal end. Instead of using empirical data, the MKM predicts the cell survival after receiving CIRT using the saturation corrected dose-mean specific energy $Z_{1D}^{*}$ deposited to a subcellular volume, i.e., a domain. Since the NIRS-MKM has to build technical continuity with the Kanai model, the biological RBE calculated by the NIRS-MKM was rescaled to the clinical RBE defined by the Kanai model by using a new clinical dose scaling factor, $F_{clin}$. Meanwhile, the LEM assumes that the biological effect of ionizing radiation on a cellular scale only depends on the mean number of killing events per cell, given by the total local energy deposition, regardless of whether the energy has been deposited by photons or ions. In further studies, many updated versions [13–16] were developed, but only the LEM was used in clinical CIRT. In vitro, in vivo, and even inpatient studies were performed to assess the LEM and MKM. However, their performances still call for verification via clinical analysis with a large number of patients [17]. The clinical dose defined by the Kanai model and NIRS-MKM and the RBE-weighted dose defined by the LEM are referred to as the RBE-weighted dose hereafter.

In 2015, our centre started clinical CIRT with an ES beam. Four years later, our centre acquired RayStation (V10B, Raysearch, Sweden). This treatment planning system (TPS) is one of the few commercially available TPSs that can perform MKM-based CIRT. Furthermore, validation of the RBE calculated by the MKM in RayStation (Ray-MKM) was necessary based on our ES beam, but relevant studies were lacking. In the literature, Fossati et al. created and validated a Monte Carlo (MC) dose engine [18, 19] simulating the ES beam at the National Center of Oncological Hadrontherapy and the RS beam at the NIRS [20]. Subsequently, they performed an RBE-weighted dose conversion study from the Kanai model to LEM [20, 21]. After that, Magro et al. coupled the NIRS-MKM with this MC dose engine [22]. Later, the parameters of the MKM were integrated into an analytical carbon-ion dose engine, namely, a fast recalculation on GPU [23]. These studies were performed on their in-house platforms.

Prior to clinical application, this study examines the Ray-MKM-calculated RBE based on our ES beam via phantom studies. We first benchmark the Ray-MKM by defining $F_{clin}$ at our centre. After that, the residual RBE difference between the Ray-MKM and NIRS-MKM is analysed. To investigate the origin of the difference, we further compare the $Z_{1D}^{*}$ differences calculated by the Ray-MKM and NIRS-MKM.

## Methods

### Modified microdosimetric kinetic model at the NIRS (NIRS-MKM)

As it represents the moderate radiosensitivity of human tumours, the NIRS-MKM chose the HSG tumour cell as the reference to calculate CIRT RBE. Based on this cell line, the RBE-weighted dose $D_{RBE} \left( {\text{x}} \right)$ at position x is calculated as follows:

1 $$\begin{aligned} D_{RBE} \left( x \right) = &\, F_{clin} {*}\left( { - \frac{{a_{r} }}{{2{*}\beta }}} \right. \\ & \left. { + \;\sqrt {\left( {\frac{{a_{r} }}{{2{*}\beta }}} \right)^{2} + \frac{{a_{0} {*}D_{Abs} \left( {\text{x}} \right) + \beta *z_{1Dmix}^{*} \left( {\text{x}} \right){*}D_{Abs} \left( {\text{x}} \right) + \beta *D_{Abs}^{2} \left( x \right)}}{\beta }} } \right) \\ \end{aligned}$$

where $D_{Abs} \left( {\text{x}} \right)$ is the absorbed dose at point x. $\alpha_{0} = 0.172\;Gy^{ - 2}$ is the initial slope of the HSG survival curve at the limit of LET = 0 [7], and $z_{1Dmix}^{*} \left( x \right)$ is the dose-mean $Z_{1D}^{*} \left( x \right)$ in a mixed-radiation field and is given as follows:

2 $$z_{1Dmix}^{*} \left( x \right) = \frac{{\mathop \sum \nolimits_{i} d_{i} \left( x \right)*z_{1Di}^{*} \left( x \right)*w_{i} }}{{\mathop \sum \nolimits_{i} d_{i} \left( x \right)*w_{i} }}$$

where $d_{i} \left( x \right)$, $w_{i}$, and $Z_{1Di}^{*} \left( x \right)$ are the absorbed dose, the relative weight, and the saturation-corrected dose mean specific energy $z_{1D}^{*}$ of the $i$th beam, respectively. To calculate the individual $z_{1D}^{*}$ of the $i$th beam, two precalculated tables are needed. The first is $z_{1D}^{*}$ as a function of the kinetic energy of ion types Z = 1–6. The table is calculated based on the amorphous track structure model [24, 25]. The model parameters, i.e., the radius of the HSG cell nucleus $\left( {R_{d} = 3.9\;\upmu {\text{m}}} \right)$ and domain $\left( {r_{d} = 0.32\;\upmu {\text{m}}} \right)$ as well as the $\alpha_{0}$ value, are determined using weighted least-squares regression based on Furusawa et al.’s in vitro studies and thus can best reproduce the relationship between HSG tumour cell survival versus the dose-averaged LET (LETd) of ^3^He-, ^12^C-, and ^20^Ne-ion beams [7]. The second table shows the fragment spectra of the carbon-ion beam, which is generated by the Monte Carlo simulation tool PTSsim [26]. This tool is based on GENT4 (version 9.2 with patch 01) [27]. The quantum molecular dynamics (QMD)-based package ‘G4QMD’ is selected for considering the nuclear reaction. $\upbeta = 0.0615\;Gy^{ - 1}$ is constant and independent of the radiation type [28]. After years of clinical applications, the NIRS suggested using carbon ions as the reference radiation instead of X-rays. The corresponding linear coefficients of the LQ model for HSG tumour cell $\alpha_{r}$ are calculated to be $0.764\;Gy^{ - 1}$ since the $z_{1Dmix}^{*} \left( x \right)$ at the centre of the representative beam is $0.963\;Gy$. The parameters of the representative beam are described in detail in the next section. A new $F_{clin} = 2.41$ was used to confirm that the clinical RBE of NIRS-MKM could reproduce the CIRT experience with the Kanai model [8]. However, this reformulation was based on the RS beam at the NIRS, although the NIRS-MKM was designed for the HS beam.

### Benchmarking the Ray-MKM

We expected the Ray-MKM to yield the same RBE as the NIRS-MKM, so that based on the same RBE-weighted prescription, patients at our centre would receive the same absorbed dose irradiation as the patients at the NIRS. However, we should note that the RBE calculation depends on the beam quality and fragment spectra, even when using the same model.

The benchmark started by configuring the Ray-MKM with the same parameters as the NIRS-MKM. After that, we followed Inaniwa et al.’s approach [8] to adjust $F_{clin}$ by using the so-called representative beam. This beam was originally generated by the RS beam at the NIRS. We simulated this beam by using our ES beam. First, we created a cube target [6.0 × 6.0 × 6.0 cm^3^, modulation width: 6.0 cm (M6)] centred at 18.12 cm [range: 21.1 cm (R21.1)] in a virtual water phantom (WP). We then generated a single-beam SOBP plan with a Ray-MKM prescription of 5.8 Gy (D5.8). This plan is abbreviated as R21.1M6D5.8. The dose distributions as well as the RBEs were compared to their counterparts reported by Inaniwa et al. [8]. The deviations of the target mean RBE were used to derive a new $F_{clin}$ for our beam and create a new Ray-MKM with it. After that, we reoptimized the SOBP plans using the new Ray-MKM and performed the comparisons again. We repeated these procedures until the target mean RBE deviation was close to zero.

### Evaluation of the Rray-MKM

After the benchmark, Inaniwa et al. also provided several dose distributions of six cube plans based on their broad beam [8] to evaluate the residual difference between the NIRS-MKM and the Kanai model. Detailed information is provided in Table 1. We also generated the same SOBP plans but with the Ray-MKM and our beamline and compared the dose distributions to the NIRS-MKM.

**Table 1:** Parameters of the evaluation plans in WPs

Energy (MeV/u)^a^	Target size (mm^3^)	Centres of SOBP (cm)	DRBE [Gy(RBE)]	Name of SOBPs
290	6.0 × 6.0 × 6.0	12.2^b^	5.8	R15.2M6D5.8^c^
350	6.0 × 6.0 × (3.0–12.0)	18.12	3.6–8.0	R21.1M(3,6,12)D5.8, R21.1M6D(3.6,5.8,8.0)
400	6.0 × 6.0 × 6.0	23.47	5.8	R26.5M6D5.8

^a^The original SOBPs were generated based on the RS beam at the NIRS, and the given energies are the energies of the monoenergetic beams; ^b^The distance from the centres of SOBPs to the surface of the WP; ^c^R: Range, M: Modulation width, D: the prescribed RBE-weighted doses

### Comparison of $Z_{1D}^{*}$ for the SOBP plans

According to Eq. (1), $Z_{1D}^{*}$ determines the RBEs and is independent of cell LQ parameters or absorbed doses. Therefore, the $Z_{1D}^{*}$ difference between the Ray-MKM and NIRS-MKM should explain the residual errors even after applying the new $F_{clin}$ for the Ray-MKM. To derive $Z_{1D}^{*}$, we rearranged the parameters in Eq. (1) and obtained Eq. (3),

3 $$Z_{1Dmix}^{*} = \frac{{\beta *\left( {\left( {\frac{{D_{RBE} }}{{F_{clin} }} + \frac{{\alpha_{r} }}{2*\beta }} \right)^{2} - \left( {\frac{{\alpha_{r} }}{2*\beta }} \right)^{2} } \right) - \alpha_{0} *D_{abs} - \beta *D_{abs}^{2} }}{{\beta *D_{Abs} }}$$

We calculated the $Z_{1D}^{*}$ in-depth for all the SOBP plans in Table 1 and compared them to the NIRS counterparts.

### RBE-weighted dose conversions to the LEM

In the literature [20, 21, 29–32], several studies converted the NIRS experience to the LEM. The NIRS-MKM purports to reproduce the CIRT experience with the Kanai model. Thus, the Ray-MKM after the benchmark should be able to reproduce the former conversion studies. Before that, the LEM RBE in RayStation was validated by referring to our clinical LEM RBE calculated by Syngo (V13C, Siemens, Germany) [33].

We first generated various SOBP plans with the Ray-MKM in WPs. The SOBP settings were described as follows: 1, the distance from the centres of SOBPs to the surface of WPs was 7.0 cm (ISO7) and 11.0 cm (ISO11); 2, the shapes of targets were cube and sphere; 3, the diameters or the dimensions of targets were from 4.0 to 12.0 cm with a step size of 2.0 cm; 4, the prescribed Ray-MKM dose was 4.0 Gy; and 5, the beam configurations were single, two opposed, and two orthogonal beams. Then, the LEM was used to recalculate the RBE-weighted dose distributions based on the Ray-MKM-optimized fluence and beam configurations. Finally, the converted LEM doses were compared to existing literature [20].

## Results

**Fig. 1:**
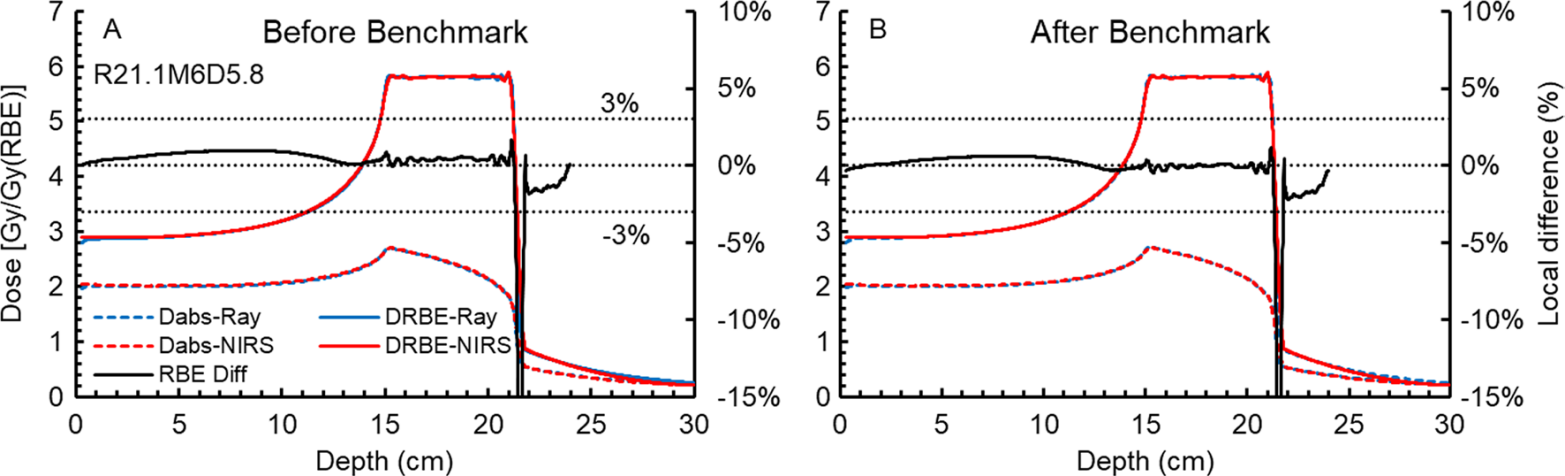
The DDDs of R21.1M6D5.8 before (**A**) and after benchmark (**B**). The blue solid and dashed lines are the RBE-weighted (D_RBE_-Ray) and absorbed (D_abs_-Ray) DDDs from the Ray-MKM, the red solid and dashed curves are RBE-weighted (D_RBE_-NIRS) and absorbed (D_abs_-NIRS) DDDs from the NIRS-MKM, and the black dashed lines are the local RBE differences

The $F_{clin}$ was determined to be 2.40. Figure 1 shows the RBE-weighted (D_RBE_-Ray) and absorbed (D_abs_-Ray) depth dose distributions (DDDs) from the Ray-MKM as well as the respective RBE-weighted (D_RBE_-NIRS) and absorbed (D_abs_-NIRS) DDDs from the NIRS-MKM before (A) and after (B) the benchmark using R211M60D5.8. The local RBE deviation curve, i.e., the black solid lines, ends when the RBE-weighted dose is < 10% of the target prescription. A deviation below that threshold would be neglected since it is not clinically relevant. The deviation of − 3–3% is marked on the figure for reference. The RBE deviation between the Ray-MKM and NIRS-MKM is within 3%. The corresponding target mean dose deviations are listed in Table 2.

**Table 2:** The target mean RBE-weighted and absorbed doses of all SOBPs

SOBP	Ray-MKM versus NIRS-MKM			
	D_RBE_^a^	D_abs_-Ray^b^	D_abs_-NIRS^c^	RBE Diff (%)
R21.1M6D5.8	5.8	2.39	2.40	0.42
R15.2M6D5.8	5.8	2.32	2.36	1.69
R26.5M6D5.8	5.8	2.47	2.47	0.00
R21.1M3D5.8	5.8	2.15	2.15	0.00
R21.1M12D5.8	5.8	2.68	2.71	1.11
R21.1M6D3.6	3.6	1.48	1.49	0.67
R21.1M6D8.0	8.0	3.31	3.33	0.60

^a^The target mean RBE-weighted dose [Gy(RBE)] of the Ray-MKM and NIRS-MKM

^b^The target mean absorbed doses from the Ray-MKM (Gy)

^c^The table indicates absorbed doses from the NIRS-MKM (Gy)

**Fig. 2:**
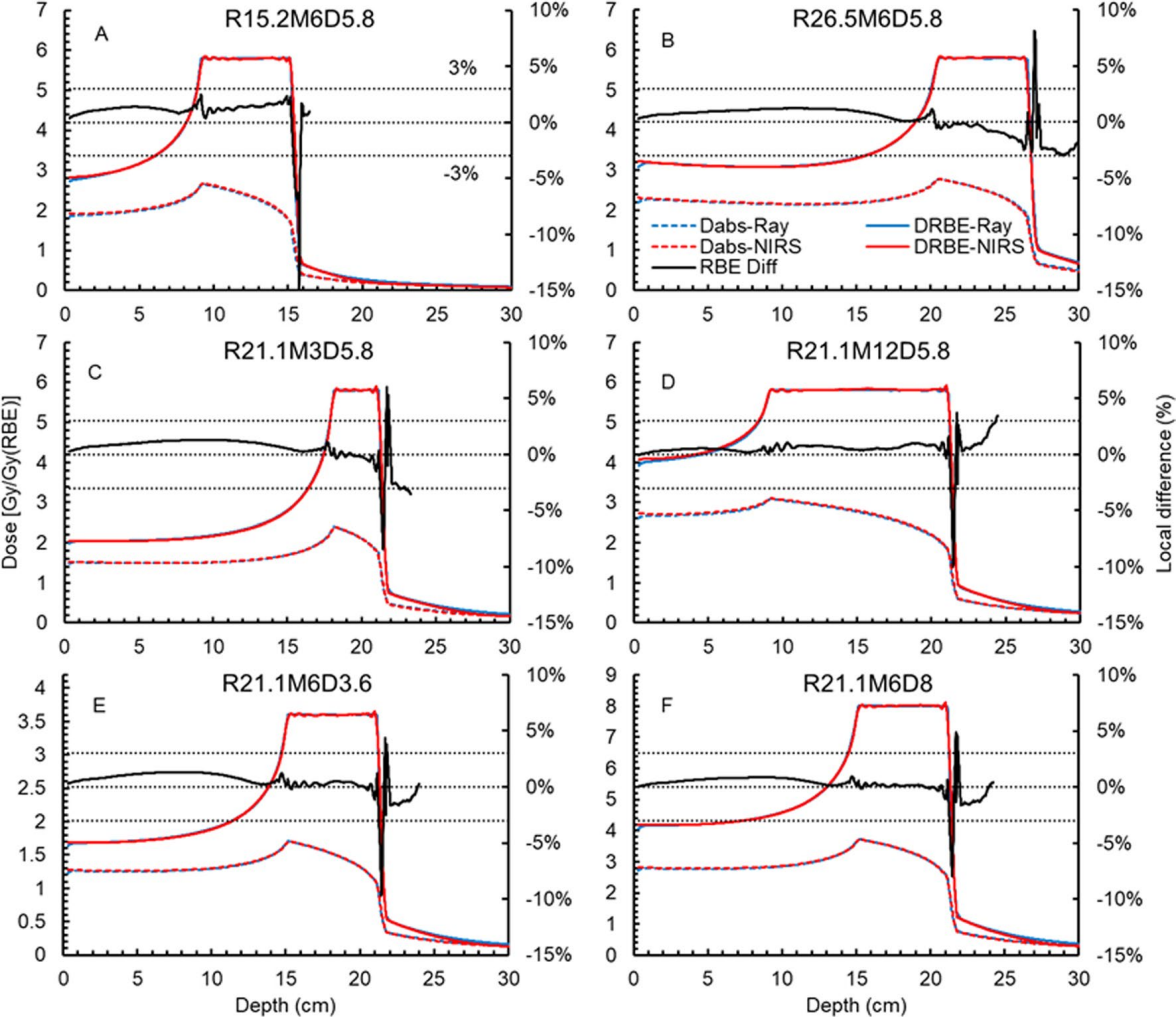
The dose cube comparisons between the Ray-MKM and NIRS-MKM. These cubes use different ranges (**A** and **B**), SOBP widths (**C** and **D**), and prescriptions (**E** and **F**)

We further compared the Ray-MKM to NIRS-MKM in terms of different ranges (Fig. 2A and B), SOBP widths (Fig. 2C and D), and prescribed doses (Fig. 2E and F). Figure 2A and B show the DDDs of R15.2M6D5.8 and R26.5M6D5.8. Figure 2C and D are R21.1M3D5.8 and R21.1M12D5.8. Figure 2E and F illustrate R21.1M6D3.6 and R21.1M6D8.0. Table 2 summarizes the target mean absorbed doses of the SOBP plans in Figs. 1 and 2. The corresponding doses from the NIRS-MKM are given for reference. The mean target RBE deviation from the NIRS-MKM is median: 0.6 (minimum: 0.0 to maximum: 1.69) %.

**Fig. 3:**
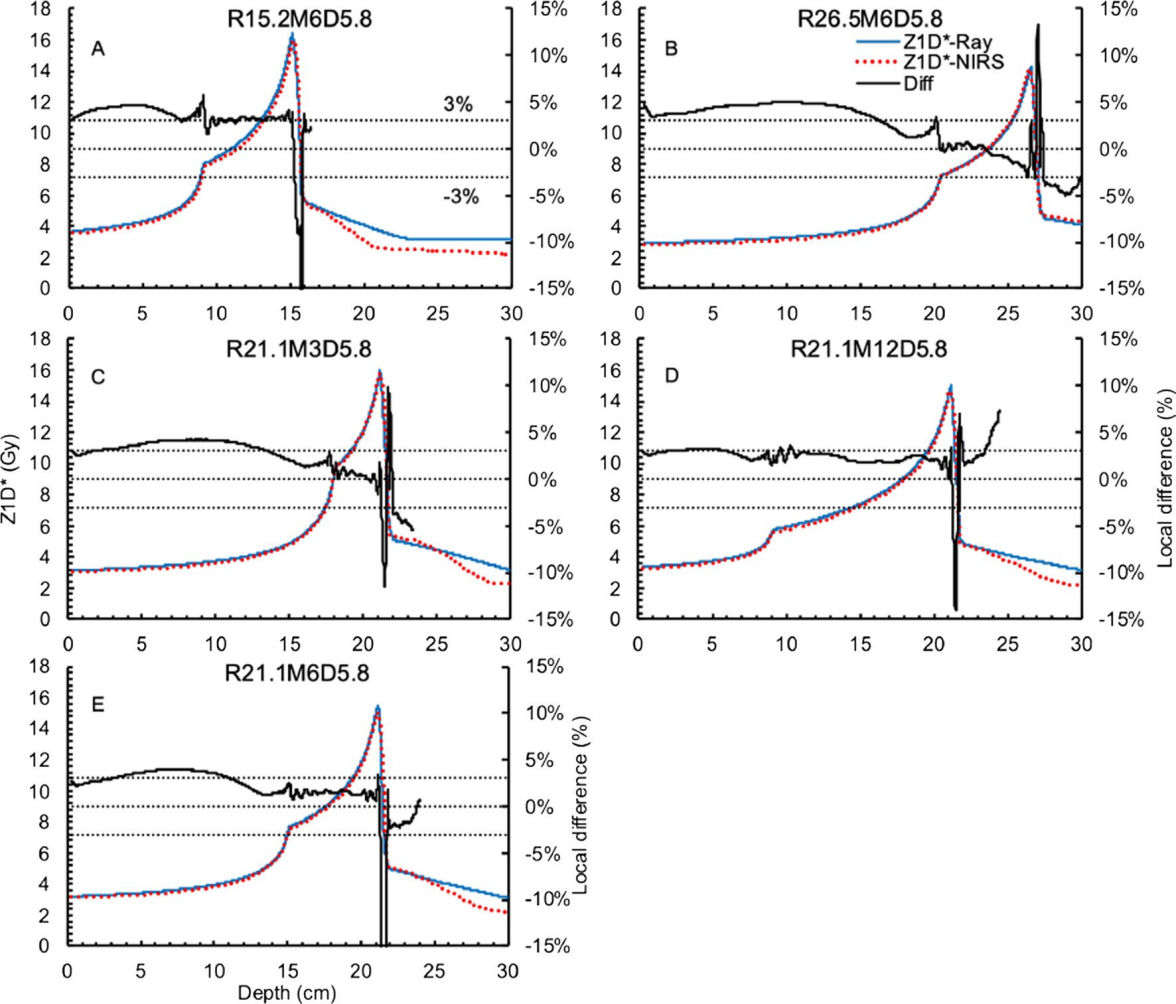
The $Z_{1D}^{*}$ in-depth of R15.2M6D5.8 (**A**), R25.6M6D5.8 (**B**), R21.1M3D5.8 (**C**), R21.1M12D5.8 (**D**), and R21.1M6D5.8 (**E**) from RayStation ($Z_{1D}^{*}$-Ray) and the NIRS ($Z_{1D}^{*}$-NIRS). The black solid lines denote the local difference between RayStation and the NIRS

Figure 3 displays the $Z_{1D}^{*}$ in-depths of all cube plans in Figs. 1 and 2. They are for R15.2M6D5.8 (A), R26.5M6D5.8 (B), R21.1M3D5.8 (C), and R21.1M12D5.8 (D). For the $Z_{1D}^{*}$ in-depths of R21.1M6D5.8 (E), R21.1M6D8.0, and R21.1M6D3.6, we only show the first one since they should share the same $Z_{1D}^{*}$. Similar to Figs. 1 and 2, the $Z_{1D}^{*}$ deviations for the RBE-weighted dose < 10% of the target prescription are not shown. The $Z_{1D}^{*}$ values from RayStation are mostly higher than those from the NIRS, which explains why the Ray-MKM needs a smaller $F_{clin}$ to maintain the same RBE as the NIR-MKM. The $Z_{1D}^{*}$ in-depth of R15.2M6D5.8 (A) shows the largest difference, i.e., approximately 3% in the target region and > 3% in the beam entrance. For the deeper-seated targets, i.e., R26.5M6D5.8 (B) and R21.1M6D5.8 (E), the deviations are smaller. The $Z_{1D}^{*}$ difference at the distal end is remarkable.

Table 3 summarizes the converted LEM doses from the Ray-MKM of 4.0 Gy (RBE). The converted LEM doses from the Kanai model in Fossati et al.’s study are listed for reference. The deviations range between − 1.8 and 0.7%, which indicates that the LEM doses converted from the Ray-MKM are comparable to those converted from the Kanai model. The converted LEM doses based on the deep-seated targets, i.e., ‘ISO11’, are closer to the reference.

**Table 3:** The converted LEM doses from the RBE-weighted dose prescription of 4.0 Gy (RBE) with the Ray-MKM

NIRS ^a^4.00	Converted LEM doses			
	Opposed beams		Orthogonal beams	
	Cube	Sphere	Cube	Sphere
*Ray-MKM*				
ISO7	4.45	4.43	4.43	4.42
	(4.29–4.53)	(4.29–4.51)	(4.28–4.52)	(4.28–4.50)
ISO11	4.45	4.44	4.44	4.43
	(4.33–4.55)	(4.31–4.53)	(4.30–4.54)	(4.30–4.52)
Reference^a^	4.50	4.40	4.50	4.45
	(4.40–4.60)	(4.30–4.50)	(4.40–4.60)	(4.30–4.55)

NIRS 4.00	Converted LEM doses	
	Single beam	
	Cube	Sphere
*Ray-MKM*		
ISO7	4.42	4.40
	(4.26–4.51)	(4.26–4.48)
ISO11	4.43	4.42
	(4.29–4.53)	(4.28–4.56)
Reference^b^	4.50	4.45
	(4.40–4.60)	(4.30–4.55)

^a^Gy (RBE); ^b^The converted LEM doses from the Kanai model

## Discussion

We first benchmarked the Ray-MKM according to the NIRS-MKM based on our ES beam. Then, we validated the residual difference between the Ray-MKM and NIRS-MKM based on an additional six cube plans. The residual errors still existed, and the largest deviation was observed in R15.2M6D5.8. To investigate the origin, we calculated the $Z_{1D}^{*}$ of all the SOBPs and compared them to those from the NIRS. After that, we validated the LEM doses converted from the Ray-MKM versus the literature.

The value of $F_{clin}$ at the NIRS changed from 1.44 in the Kanai model to 2.41 in the NIRS-MKM. By using 1.44, CIRT with the Kanai model could refer to the NIRS’s experience with neutron therapy, that the clinical RBE at the neutron-equivalent position was 3.0 when the total fractionation was 16 and the corresponding absorbed dose per fraction at that position was 0.9 Gy [5]. The neutron-equivalent position is the depth where the LETd is $80\;{\text{keV}}\;um^{ - 1}$ at a carbon-ion SOBP of 290 MeV u^−1^. After years of experience with CIRT, the NIRS was determined to use carbon ions as the reference radiation, and the NIRS-MKM was applied to calculate the RBE for the scanning beam. To build technical continuity with the Kanai model, the NIRS-MKM matched the clinical RBE at the new reference position, i.e., at the centre of R21.1M6D5.8 [8]. This is because the calculated absorbed dose at the position could result in the same in vitro HSG tumour cell response as that of the Kanai model. The radiation quality at this position may be minimally affected by the delivery technique. In this study, we also simulated the representative beam based on our beamline and compared it to the NIRS counterpart. The difference between them was small. Therefore, we could expect the Ray-MKM to generate the same RBE as the NIRS-MKM. Furthermore, benchmarking the Ray-MKM in the same way as the NIRS-MKM could connect our modelled RBE to the NIRS clinical experience.

However, the different delivery systems could create notable RBE differences. The reformulation of CIRT RBE at the NIRS was based on their broad beam. Fossati et al.’s study [20] showed that the RBE of the active-scanning beam would be slightly higher than the RBE of the broad beam, especially for shallow targets. Similar results can also be found in Inaniwa et al.’s study [10]. Based on their results, to maintain the same RBE, the active-scanning beamline may need to use a smaller $F_{clin}$. In this study, our benchmark determined the $F_{clin} = 2.40$ for the Ray-MKM. Similarly, Magro et al. also performed a study with their active dose system. The $F_{clin}$ they determined was 2.39 [22].

It seems that the $Z_{1D}^{*}$ values based on our beamline were mostly higher than those from the NIRS counterparts [7], and the difference at the distal end was more remarkable. This may be due to the beam quality and fragment spectra. First, as we mentioned before, different delivery techniques played a role. In addition to Fossati et al.’s study, Inaniwa et al. simulated the DDDs and RBEs of three SOBPs delivered by the RS, ES, and HS beams [10]. They concluded that compared to the ES beam and HS beam, RS increased the multiscattering and nuclear reactions, thus degrading the beam quality, which was especially pronounced for shallow targets. Their results could explain the $Z_{1D}^{*}$ deviations within the entrance and targets for the SOBPs in this study. Furthermore, $Z_{1D}^{*}$ was calculated based on the fragment spectra, which were previously generated by Monte Carlo simulations [19]. The accuracy of the simulation to measurements may be a source of uncertainty. Our previous study demonstrated that the fragment spectra in RayStation overestimated the LET distally compared to the fragment spectra in Syngo [34]. Moreover, the different types of MC toolkits may be another reason. FLUKA [19] and GEANT4 with ‘G4GMD’ [7] were used to generate the fragment spectra for our system and the NIRS-MKM, respectively. A comparison between FLUKA (version 2008.3) and GEANT4 (version 9.3) illustrated that discrepancies > 10% could be found for nuclear fragments [35]. Thus, different toolkits may amplify the differences, especially at the distal end, where the doses were solely contributed by the nuclear fragments.

It is true that the RBE calculation depends on the beam quality and fragment spectra, even when using the same model. However, it seems that the radiation quality of a deep-seated target, i.e., R21.1M6D5.8, can be minimally affected by all of the factors we discussed before. Therefore, the same RBE can be expected. Furthermore, although each facility may have different radiation characteristics and different approaches for simulating the beamline, it is good to determine their centre-specific $F_{clin}$ based on this approach; thus, patients at different centres could receive the same prescribed dose of irradiation connected to the CIRT experience at the NIRS.

Compared to the proton beam, the carbon-ion beam has a higher nuclear cross-section when interacting with human tissue, thus creating a central, large angle, low intensity, and non-Gaussian lateral dose halo. The problem created by this halo is that it removes nonneglectable doses from the central Gaussian region. Therefore, accurately modelling the halo is important for predicting either absorbed or RBE-weighted doses. The NIRS-MKM adopted a trichrome approach [11, 36] for both RBE-weighted and absorbed dose calculation. Three Gaussian components considered the primary carbon ion, heavy fragments with Z ≥ 3, and light fragments with Z ≤ 2. Meanwhile, RayStation applied a similar but more complicated five-Gaussian approach for absorbed dose calculation. Similarly, the first components represented primary carbon ions, while the second and the rest of the three Gaussians represented heavier fragments and lighter fragments, respectively. For RBE-weighted dose calculation, RayStation adopted a monochrome approach, similar to the Kanai model and LEM.

The NIRS treated many more patients than any other single CIRT centre. Their experience was valuable for the whole CIRT community. Therefore, many studies have aimed to convert the NIRS experience, i.e., experience with the Kanai model, to the LEM. As a part of validation, this study confirmed that the Ray-MKM was capable of reproducing the conversion study. However, we should be cautious that the RBE-weighted doses based on even the same absorbed dose between the Kanai model and the NIRS-MKM had a notable difference in the beam entrance and distal end [8]. Consequently, the organs at risk constraints based on the Kanai model may not be applicable for treatment planning with the NIRS-MKM.

The $Z_{1D}^{*}$ as a function of kinetic energy could be verified by using an analytical approach using MATLAB (Math Work Inc. USA), which was described in Margo et al.’s study [22]. This table in RayStation was provided by our vendor but certified by the NIRS. We did not verify this table since Margo et al.’s verification indicated that the small discrepancies did not affect the agreement with the NIRS-MKM. The accuracy of the fragment spectra could be alternatively validated by using an ion track detector, i.e., CR-39 [37]. The conversion studies only used the RBE-weighted prescriptions of 4.0 Gy (RBE). In clinics, many more prescriptions at approximately 4.0 Gy (RBE) were used. This study did not involve a patient study, which referred to the performance of dose calculation within the inhomogeneity. However, this pencil beam dose engine has been commissioned. The unpublished data showed that the absorbed dose deviation within inhomogeneity was smaller than 1%.

## Conclusion

We validated the Ray-MKM based on our ES beam through several SOBP plans. A benchmark was performed to minimize the RBE difference between the Ray-MKM and NIRS-MKM. To investigate the origin of the RBE difference, we calculated the $Z_{1D}^{*}$ in depth of all the SOBP plans and compared it to the NIRS counterparts. The $Z_{1D}^{*}$ difference was more pronounced for the shallow targets, which may be due to more severe differences caused by the radiation quality and fragment spectra. For the $Z_{1D}^{*}$ deviations at the distal end, since the absolute dose deviations were very small, we ignored the differences. Furthermore, although each facility may have different radiation characteristics and approaches for simulating the beamline, it may be good to determine its centre-specific $F_{clin}$ based on this approach; thus, patients at different centres could receive the same prescribed dose of irradiation connected to the CIRT experience at the NIRS.

## Data Availability

The datasets used and analysed during the current study are available from the corresponding author on reasonable request.

## Abbreviations

RBE: Relative biological effectiveness
Ray-MKM: The modified microdosimetric kinetic model in RayStation
CIRT: Carbon-ion radiotherapy
SOBP: Spread-out Bragg-peak
NIRS: The national institute of radiobiological science in Japan
RS: Range shifter scanning
ES: Active-energy scanning
HS: Hybrid scanning
NIRS-MKM: MKM at NIRS
LEM dose: The RBE-weighted doses with local effect model I
Kanai model: The linear quadric model at NIRS
LET: Linear energy transfer
HIMAC: Heavy ion medical accelerator at Ciba
TPS: Treatment planning system
MKM dose: The RBE-weighted dose with MKM
$Z_{1D}^{*}$: Saturation-corrected dose-mean specific energy
$F_{clin}$: Clinical dose scaling factor
WP: Water phantom
DDD: Depth dose distribution
D_abs_-Ray: Absorbed doses in RayStation
D_RBE_-Ray: RBE-weighted doses in Ray-MKM
D_RBE_-NIRS: RBE-weighted doses at NIRS
D_abs_-NIRS: Absorbed doses at NIRS
LETd: Dose averaged LET
$Z_{1D}^{*}$-Ray: $Z_{1D}^{*}$ In RayStation
$Z_{1D}^{*}$-NIRS: $Z_{1D}^{*}$ From NIRS
